# *Prevotella copri* variants among a single host diverge in sphingolipid production

**DOI:** 10.1128/mbio.02409-23

**Published:** 2024-01-18

**Authors:** Xieyue Xiao, Henry H. Le, Min-Ting Lee, Daniel Lamm, Elizabeth L. Johnson, Ilana L. Brito

**Affiliations:** 1 Meinig School of Biomedical Engineering, Cornell University, Ithaca, New York State, USA; 2 Division of Nutritional Sciences, Cornell University, Ithaca, New York State, USA; 3 Howard Hughes Medical Institute, Chevy Chase, Maryland, USA; Northern Arizona University, Flagstaff, Arizona, USA

**Keywords:** *Prevotella*, sphingolipids, microbiome, host–microbe interactions, *Prevotella copri*

## Abstract

Sphingolipids serve as vital structural and signaling components of the cell membranes in both eukaryotes and prokaryotes. Within the gut microbiome, *Bacteroides* species have been identified as major producers of sphingolipids, and *Bacteroides*-produced sphingolipids have been shown to be modulators of host immune and metabolic functions. While *Bacteroides* species are a prominent feature of the gut microbiomes of populations living in industrialized countries, *Prevotella copri*, a member of the same phyla, albeit a different family, is the dominant feature across the remainder of the global population, although their sphingolipid-producing capabilities have not been as thoroughly investigated. To fill this gap, we examined the genomes of over 60 diverse isolates of *P. copri* and identified several key enzymes involved in sphingolipid synthesis in *P. copri*. Combining bioorthogonal labeling and liquid chromatography-mass spectrometry (LC-MS) based lipidomics, we functionally characterized the first step in *P. copri de novo* sphingolipid synthesis in addition to profiling the sphingolipidomes of *P. copri* strains, identifying key enzymes that may play roles in producing a diverse set of *P. copri* sphingolipids. Given the limited genetic engineering tools amenable for use in *P. copri*, our approach takes advantage of comparative genomics and phenotypic profiling to explore sphingolipid production in these understudied, yet highly prevalent, organisms.

**IMPORTANCE:**

Sphingolipids are important signaling molecules for maintaining metabolic and immune homeostasis in the host. These lipids are also produced by gut commensals, most notably by *Bacteroides* species. Despite the global prevalence of *Prevotella copri* in gut microbiomes of individuals, little is known about the types of sphingolipids they produce and whether they are similar in composition and structure to those produced by *Bacteroides*. Given the varied associations of *P. copri* with diverse sphingolipid-related health outcomes, such as rheumatoid arthritis and glucose intolerance, it is important to first characterize the specific sphingolipids produced by individual strains of *P. copri* and to identify the genes involved in their pathways of production. This characterization of *P. copri*-derived sphingolipids provides further insight into how bacterial sphingolipid production can serve as a mechanism for microbial modulation of host phenotypes.

## INTRODUCTION

Sphingolipids are a unique class of bioactive lipids known to engage in multiple processes including subcellular trafficking (1), cell proliferation, central nervous system development (2), and immunity (3, 4). The manipulation or use of host sphingolipids by microbial pathogens promotes their pathogenicity and infectivity (5–7). Although sphingolipids are best understood as a product of eukaryotic synthesis, a subset of bacteria also produce sphingolipids, including members of the Bacteroidota (formerly known as Bacteroidetes) phylum, one of the most prevalent phyla worldwide, and often constitute a significant portion of the gut microbial community together with Firmicutes (8, 9). Microbiota-produced sphingolipids are not only important for the fitness of bacteria (10) but are also known to improve colonic integrity (11), reduce hepatic lipid accumulation (12), and contribute to immune homeostasis (3, 13, 14) within the host.

Among the Gram-negative Bacteroidota, members of the Bacteroidaceae family are well-studied for their roles in producing a diverse set of sphingolipids (11, 12, 14, 15). A second family, the Rikenellaceae, including the less populous *Alistipes* species, have been found to produce lipids similar in structure to sphingolipids, sulfonolipids (16). *Porphyromonas gingivalis,* a member of another family known to produce sphingolipids, the Porphyromonadaceae, has recently been found to produce a set of novel sphingolipids (17). A fourth family, Prevotellaceae, is known to produce sphingolipids and is the most widely prevalent and abundant of the Bacteroidaceae due to its dominance in the gut microbiomes of the population of non-industrialized or non-Westernized countries (18). *P. copri*, the predominant member of Prevotellaceae, has also been linked to inflammatory diseases such as rheumatoid arthritis (19–21), inflammation in patients with human immunodeficiency virus (HIV) infection (22), and low-grade systemic inflammation (23). Given the immunomodulatory role of *Bacteroides-*derived sphingolipids (3, 11, 13), we sought to further characterize the sphingolipid-producing traits of *Prevotella*. Analysis via two-dimensional thin-layer chromatography supports the notion that *Prevotella* can produce sphingolipids (24, 25), and LC-MS data confirm that *P. copri* produces sphingolipids (26), including inositol sphingolipids (27), a unique class of lipids known to play signaling roles in high-order organisms, including humans (28, 29).

One reason for our limited knowledge of *P. copri*-derived sphingolipids is the lack of diverse publicly available isolates. We obtained 63 additional isolates of *P. copri* by plating gut microbiome samples obtained from study participants from the Fiji Community Microbiome Project (FijiCOMP) (30) on Medium 10 (M10) agar adjusted from previous studies (31, 32). Interestingly, these were derived from a single participant, whose microbiome was largely composed of members of the *Prevotella* genus (68 %). While *P. copri* is not readily amenable to genetic engineering, instead, this diverse set of isolates afforded us the opportunity to investigate the contributions of individual genes in the sphingolipid synthesis pathway through comparative genomics. First, we computationally identified the genes involved in the first committed steps of sphingolipid biosynthesis, including key enzymes that initiate sphingolipid synthesis. Next, using LC-MS, we confirmed that the *P. copri* isolates are capable of producing sphingolipids, including many that are similar to those previously identified as products of *Bacteroides* sphingolipid synthesis, as well as several uncharacterized sphingolipids that are potentially novel. Given the near ubiquity of *Prevotella* within the human gut microbiome, the characterization of these novel sphingolipid variants highlights the vast landscape of potential host–microbiome lipid-mediated interactions.

## RESULTS

### Serine palmitoyltransferases are identified within *P. copri* genomes

**Fig 1:**
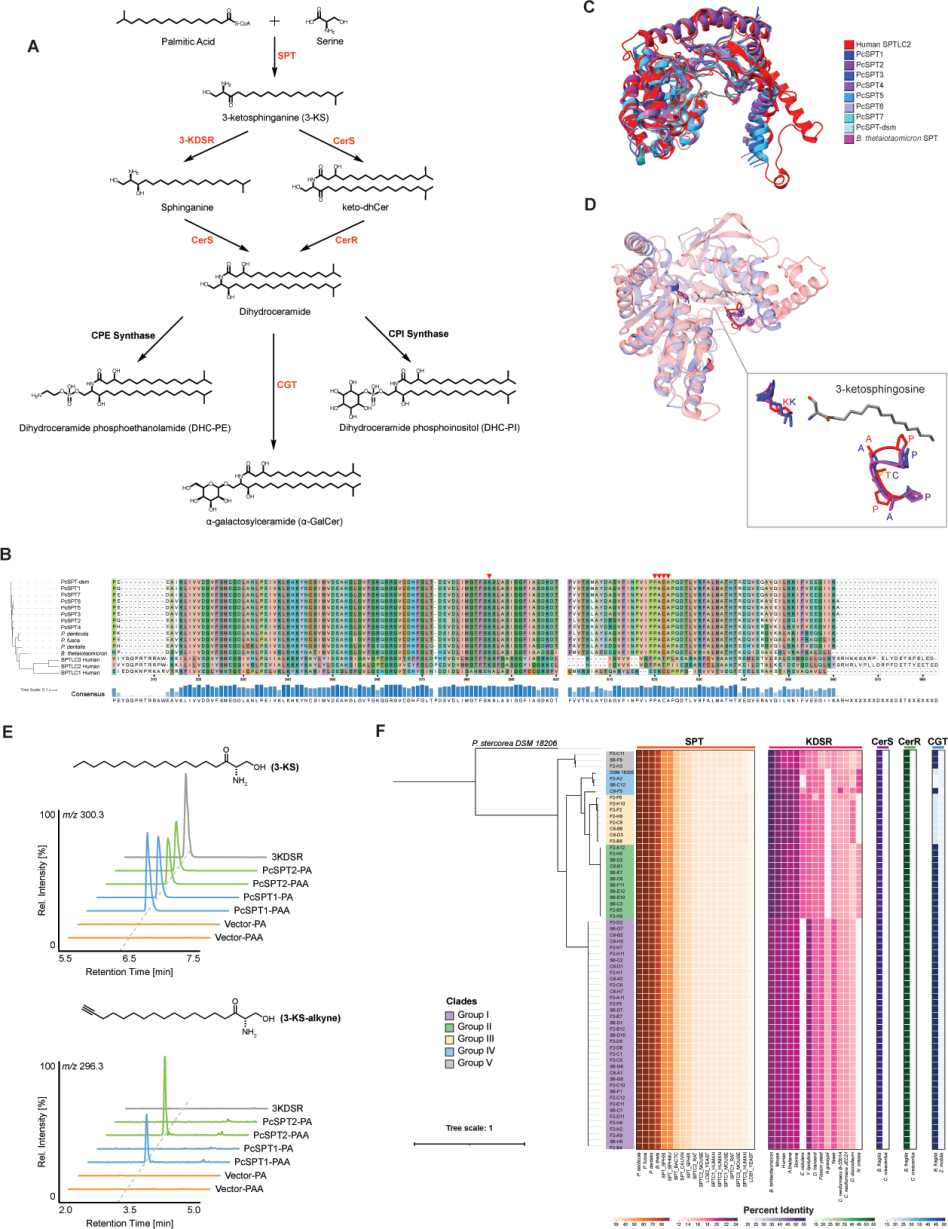
Identification and investigation of enzymes involved in sphingolipid synthesis in *P. copri*. (**A**) Schematic of potential bacterial sphingolipid biosynthesis pathways. (**B**) Multi-sequence alignment of eight SPT variants identified from *P. copri* strains and known SPT genes from other closely related bacterial species or humans. Percent identity was calculated by Clustal Omega. (**C**) Structural alignment of the eight *P. copri* SPT (PcSPT) variants predicted by ESMFold to *Bacteroides thetaiotaomicron* SPT and human SPTLC2. (**D**) Alignment of PcSPT1, *B. thetaiotaomicron* SPT, and human SPTLC2. Highlighted regions indicate the active site, Lys379, and the ligand recognition motif, either the Pro–Ala–Thr–Pro motif in human SPTLC2 or the Pro–Ala–Cys–Ala motif in *P. copri* and *B. thetaiotaomicron*. The box on the right shows a zoomed-in view of the active site and its ligand, 3-KS. Characters denote the amino acids of the motifs in human SPTLC2 (red) or in *P. copri* and *B. thetaiotaomicron* (blue). (**E**) Ion chromatograms representing the detection of 3-KS or 3-KS-alkyne from *E. coli* BL21 heterologously expressing PcSPT1, PcSPT2, or vector control and treated with either palmitic acid (PA) or palmitic acid alkyne (PAA). (**F**) Percent identities of the SPT, 3-KDSR, CerS, CerR, and CGT enzymes predicted from *P. copri* strains to known homologous proteins from other species. The phylogenetic tree based on PhyloPhlAn marker genes shows the distance between *P. copri* isolates used in this study. Colors indicating different clades that the isolates were clustered into.

The first committed step in sphingolipid biosynthesis requires the activity of serine palmitoyltransferase (SPT), an enzyme which fuses serine to palmitoyl-coenzyme A to produce 3-ketosphinganine (3-KS) (33) (Fig. 1A). The function and structure of SPT enzymes have been studied in other species, from bacteria to humans (33). To identify SPT-like genes from the *P. copri* genomes, we searched homologous protein sequences to 19 known SPT enzymes in the genomes of the 63 *P*. *copri* isolates and the type strain, *P. copri* DSM 18205, using BLASTp (34), identifying one SPT candidate per genome. These were further grouped into seven similar yet distinct SPT gene variants, (PcSPT1–7) from the isolates and one from the strain DSM 18205 (PcSPT-dsm) with 80%–85% nucleotide similarity (Table S1).

We then compared the eight putative *P. copri* SPT gene variants to SPT enzymes from closely related *Bacteroides* and oral *Prevotella* species as well as the three human SPT enzymes. The multiple-sequence alignment (MSA) of these variants shows sequence conservation of SPT genes across species, as reported elsewhere (33) (Fig. 1B), with variability mostly located at the N- and C-terminals of the protein-coding sequence. Lys379, found in the active site in human SPTLC2, though not human SPTLC1, as SPTLC1, unlike SPTLC2, is not able to bind the cofactor pyridoxal 5’-phosphate (PLP) (35). Lys379 is present within all examined *P. copri* SPT enzymes. A Pro–Ala–Thr–Pro (PATP) motif in the tunnel formed by human SPTLC2 is suggested to be crucial for catalysis due to its specific binding of the long acyl chain of palmitoyl-CoA/3-KS (35). Interestingly, for SPT candidates we identified in *P. copri* and other bacterial genomes, the ligand recognition site appears instead as the Pro–Ala–Cys–Ala (PACA) motif (Fig. 1B through D). The molecular replacement of threonine to cysteine may enable additional redox biology in SPT for sphingolipid-producing bacteria (36). Nevertheless, the structures of these two highly similar motifs illustrate conservation between the SPT enzymes we identified from *P. copri* and other prokaryotic and non-prokaryotic species (Fig. 1C).

To validate whether our putative *P. copri* SPT variants were competent for sphingolipid production, we heterologously expressed two representatives, PcSPT1 and PcSPT2, in BL21 *Escherichia coli,* a bacterium which does not produce sphingolipids natively(5). We then cultured the transformed *E. coli* with either palmitic acid (PA), a sphingolipid precursor, or its analog, palmitic acid alkyne (PAA) (12), which can act as a surrogate for PA and is easily distinguished via LC-MS analysis. Indeed, following lipid extraction, LC-MS analysis identified production of 3-KS (via PA), or 3-KS-alkyne (via PAA), respectively, the corresponding metabolic signatures indicative of SPT activity, features which were absent in our vector control (Fig. 1E). This further suggests that there is some sequence variability tolerated in the binding pocket of the acyl chain of palmitoyl-CoA/3-KS.

### Additional *P. copri* enzymes predicted to be involved in sphingolipid synthesis

The reactions and pathways involved in the metabolism of sphingolipids have been characterized in many other species including both eukaryotes, including humans, and prokaryotes, such as *Bacteroides, Porphyromonas,* and *Sphingobacterium*(5, 9). We attempted to predict enzymes involved in steps downstream of SPT in sphingolipid synthesis in *P. copri* using both sequence and structural homology-based approaches.

Previous studies have identified two distinct pathways of bacterial sphingolipid synthesis from 3-KS (26, 37). As illustrated in Fig. 1A, one pathway involves 3-ketosphinganine reductase (3-KDSR), catalyzing the conversion of 3-KS to sphinganine, followed by the reaction catalyzed by ceramide synthase (CerS), which converts this precursor into dihydroceramides (38). The second pathway instead starts from the production of 3-keto-4,5-dihydro-ceramide (keto-dhCer) from 3-KS by CerS, followed by conversion of keto-dhCer to dihydroceramides by ceramide reductase (CerR), the crucial step in the production of more complex sphingolipids.

To build the sphingolipid synthesis pathways in *P. copri*, reviewed sequences from the UniProt database and reported sequences in previous studies of the enzymes described previously were used as references (26, 37, 39). Using BLASTp, we found homologous proteins to all these enzymatic proteins from all *P. copri* strains. The best hits to references sequences from *Bacteroides* species are above 50% identity of CerS and CerR and 20%–25% for 3-KDSR (Fig. 1F).

Ceramide galactosyltransferase (CGT) is the key enzyme for the biosynthesis of α-galactosylceramide (α-GalCer) (40), which has been shown to enable *Bacteroides fragilis* to modulate the host immune system by involving in the activation of natural killer T (NKT) cells (3, 13). Even though we did not detect α-GalCer in the two *P. copri* strains examined, we attempted to explore the potential of α-GalCer production by predicting CGT from the *P. copri* genomes. By searching known CGTs from UniProt databases and a previous study (41) using BLASTp, we found sequences with over 50% identity to *B. fragilis* CGT (BF9343_3149) in 53 of the *P. copri* genomes we analyzed and with 25% identity in the remaining genomes (Fig. 1F).

### *P. copri* DSM 18205 produces a variety of sphingolipids

After confirming the production of 3-KS, the first dedicated metabolite of sphingolipid biosynthesis, we wished to further characterize the remaining *P. copri* sphingolipidomes. To accomplish this, we used myriocin, a well-characterized inhibitor of SPT (42). Upon myriocin treatment, we expect selective reduction of sphingolipid production, a metabolic feature that can be identified via routine comparative metabolomics workflows (12, 43).

**Fig 2:**
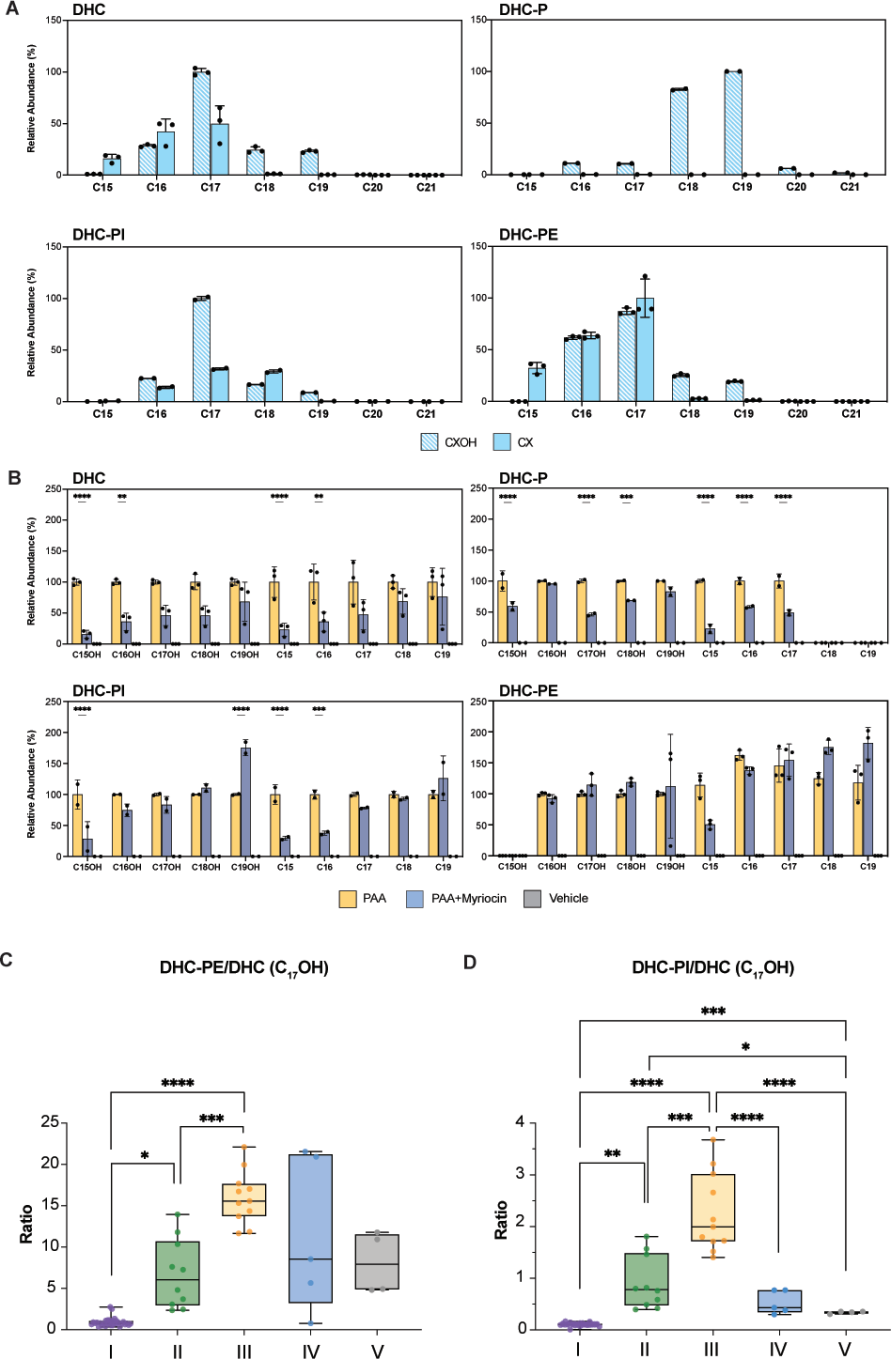
Sphingolipidomes of *P. copri* and differential sphingolipid production across strains. (**A**) Bar plots showing the relative abundances of four classes of sphingolipids of various fatty acid chain lengths detected from *P. copri* DSM 18205 by LC-MS. Colors showing hydroxylated/unhydroxylated forms. Relative abundances of metabolites were determined from the area under the curve (AUC) of peaks from LC-MS extracted ion chromatograms. Error bars indicating mean ± SD (*n* = 3). CX: non-hydroxylated sphingolipids, CXOH: hydroxylated sphingolipids. X represents the total number of carbons in the molecule. (**B**) Bar plots showing the difference in relative abundance of different sphingolipids upon myriocin treatment starting at the early log phase. Relative abundances were scaled to the non-treated group. Multiple *t*-test with Bonferroni correction for (**A**) and (**B**), **: *P* ≤ 0.01; ***: *P* ≤ 0.001; ****: *P* ≤ 0.0001. Error bars indicating mean ± SD (*n* = 3). (**C-D**) Box plots showing the abundance ratio of detected DHC-PE/DHC (**C**) and DHC-PE/DHC (**D**) from different *P. copri* isolates calculated from LC-MS results. Dots over the box plot denote the replicates of isolates (*n* = 2). Welch’s ANOVA tests were performed to investigate the differences among groups (*P* value = 8.1323e-09 for DHC-PE/DHC ratios and 5.623e-07 for DHC-PI/DHC ratios). Stars show the significance from Games–Howell tests (*: *P* ≤ 0.05; **: *P* ≤ 0.01; ***: *P* ≤ 0.001; ****: *P* ≤ 0.0001). Phylogenetic clusters were determined using PhyloPhlAn 3.0.

To initiate our analysis, we first determined if myriocin would impart growth defects on *P. copri* DSM18205. Indeed, our results showed a reduction in growth when myriocin was added immediately prior to subculturing when the myriocin concentration is higher than 1 µM (Fig. S1A), suggesting that myriocin can inhibit the growth of *P. copri* and that sphingolipid production is required for maximal fitness. For the subsequent experiments, we treated the bacterium at the early log phase to reduce effects due to changes in growth rates (Fig. S1B). As with our previous analysis, we again used PAA as a metabolic label, further ensuring that metabolic features we characterize are palmitic acid-derived. Finally, for this analysis, we utilized LC-based high-resolution mass spectrometry, enabling both superior comparative analysis and chemical formula identification, further ensuring appropriate sphingolipid identification. Therefore, a curation of myriocin-responsive, PAA-labeled, high-resolution features identified alkyne variants of sphinganine (SA), dihydroceramide (DHC), dihydroceramide phosphoethanolamide (DHC-PE), and dihydroceramide inositol (DHC-PI) (Fig. 2A and B), in both hydroxylated (CXOH) and non-hydroxylated (CX) forms, similar to those previously identified as products produced by *Bacteroides* species(3, 11, 12, 27). The major alkyne-bearing DHCs have N-attachments of either C17, C16, and C15 fatty acids, with and without beta hydroxylation of the fatty acid. Longer-chain N-acylations were also detected, albeit as minor products comparatively (Fig. 2A).

### Differential production of sphingolipids among *P. copri* isolates

Enabled by our capacity to identify sphingolipids in *P. copri,* we then wished to extend our sphingolipidomic analysis of *P. copri* variants. The human *P. copri* isolates and *P. copri* DSM18205-type strain used in this study can be phylogenetically clustered into five distinct groups (Groups I to V) based on a set of 400 marker genes (Fig. 1F; Table S2). Using our approach (*vida supra*), we then opted to profile the production of the most abundant (and therefore easiest to detect for all head groups) C17OH sphingolipids from 40 of our *P. copri* isolates that cover all five groups (Fig. S1C). Interestingly, we noted the accumulation of either DHC or DHC-PE/PI in each of the 40 isolates characterized, suggesting that some strains preferably accumulate DHC, while others continue sphingolipid biosynthesis producing more complex sphingolipids such as DHC-PE and DHC-PI (Fig. 2C and D, Fig. 1A). For example, isolates from Group I and Group III have an average nucleotide identity (ANI) of around 83%; however, the sphingolipidomic analysis shows that Group III isolates preferentially convert their C17OH to DHC-PE/PI, whereas Group I isolates accumulate DHC, highlighting divergent sphingolipid strategies among related strains (Fig. 2C and D).

### Novel sphingolipids produced by *P. copri* DSM 18205

**Fig 3:**
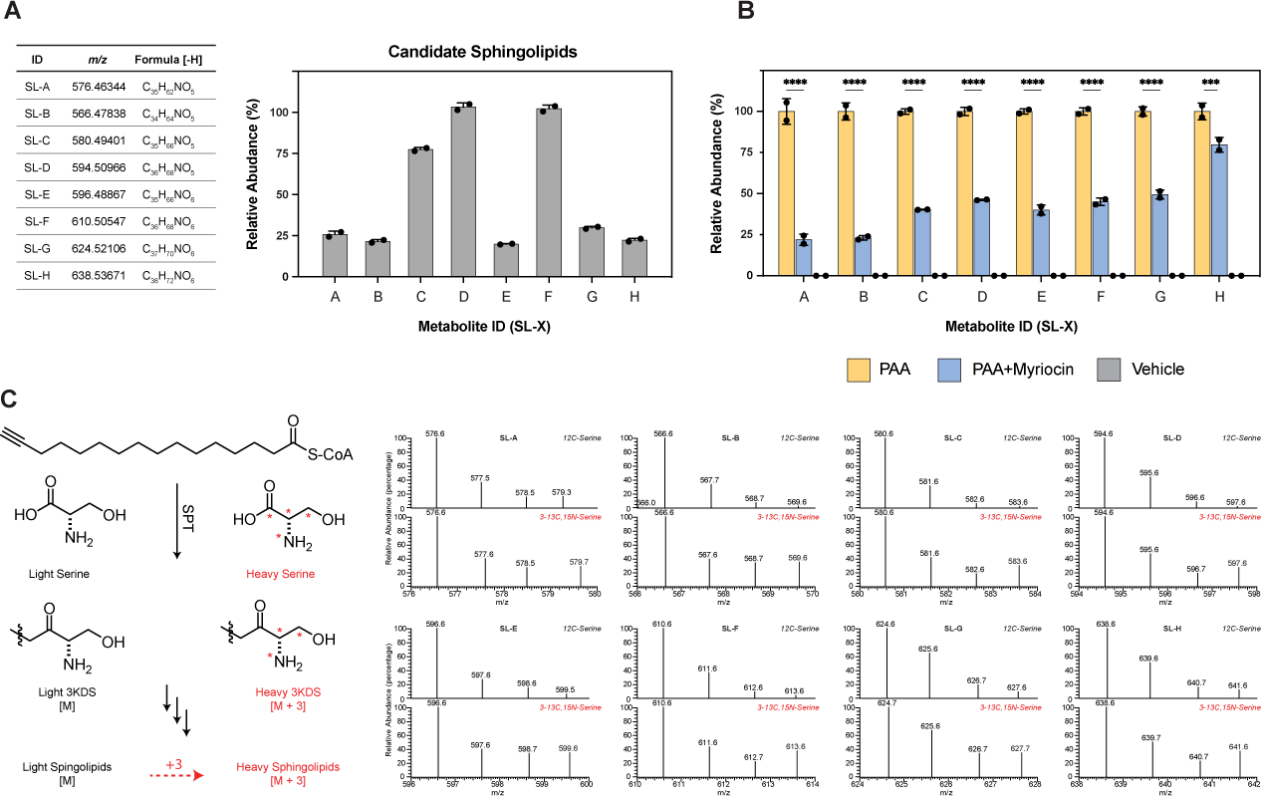
Sphingolipid candidates identified from the *P. copri* type strain. (**A**) The metabolite ID, *m/z*, chemical formulae, and relative abundances of myriocin-responsive molecular features detected by LC-MS from *P. copri* DSM 18205. Relative abundances were scaled to the molecule with the highest intensity. (**B**) The relative abundance of candidate sphingolipids with corresponding *m/z* with and without myriocin treatment (multiple *t*-test with Bonferroni correction, ***: *P* ≤ 0.001; ****: *P* ≤ 0.0001). Error bars indicating mean ± SD (*n* = 2). (**C**) Schematic and MS spectra of candidate sphingolipids from *P. copri* DSM 18205 cultures treated with either ^12^C or 13C3,15N-L-serine and PAA. Incorporation of serine in the synthesis increased the molecular mass of the metabolite by 3 when 13C3,15N-L-serine (heavy serine) is used during bacterial cultivation, as shown by the schematic in the left. In the bottom panel of each MS spectrum plot, the first and last peak represent the light and heavy sphingolipids, respectively. An increased relative amount of heavy form indicated serine as a substrate of synthesis of the metabolite.

In addition to detecting known sphingolipids produced by *P. copri* DSM18205, comparative metabolomics identified eight myriocin-responsive metabolites bearing the alkyne label, which ionize exclusively in the negative detection mode (Fig. 3A and B). Analysis of their corresponding chemical formulae suggests a group of sphingolipids which have not been previously characterized. To further validate whether these molecular features were, in fact, sphingolipids, we carried out isotopic labeling. As canonical sphingolipids bear a molecular region originating from serine, we cultured *P. copri* DSM 18205 with and without isotopically enriched13C3,15N-L-serine. To this end, we anticipate that, if our unidentified myriocin-dependent features undergo serine labeling, we clarified whether or not our novel features are sphingolipids. As anticipated, the eight putative sphingolipids were labeled with 13C2,15N (Fig. 3C), further indicating that these eight uncharacterized metabolites likely represent novel sphingolipids. Interestingly, these molecular features are not found in *Bacteroides* cultures, suggesting that these uncharacterized sphingolipids are unique to *P. copri* and therefore merit further analysis (15, 17).

## DISCUSSION

This study furthers our appreciation for *Prevotella-*derived sphingolipids within the human gut microbiome. We identified genes involved in sphingolipid production in *Prevotella* that have been conserved across bacteria. Through the use of sphingolipid synthesis inhibitors, bioorthogonal labeling of sphingolipids, and LC-MS-based comparative lipidomics, we have profiled the sphingolipidome of *P. copri*, showing their ability to produce simple and complex sphingolipids, some of which overlap with those produced by *Bacteroides*. These data highlight the overall conservation of sphingolipid synthesis pathways within Bacteroidota. However, variations in sphingolipids observed within the *Prevotella* isolates cluster according to the phylogenetic distribution, suggesting that these organisms have recently diverged with respect to specific sphingolipid production pathways.

The diversity of sphingolipids comes from variable head groups and fatty acid chain lengths. The amount of strain-level diversity in sphingolipid production is striking. Interestingly, the strains examined in this paper, save for the type strain, were all derived from a single individual’s gut microbiome. Co-existing *P. copri* strains in the gut microbiome are likely to provide different types of sphingolipids to the same host, raising an interesting question of whether strain-level diversity impacts sphingolipid-related metabolism and immune phenotypes.

Specifically, α-GalCer, produced by *Bacteroides* species, has immunomodulatory effects on the host, via the activation of invariant natural killer T (iNKT) cells (3, 13). We find CGT, the enzyme that catalyzes the production of α-GalCer, in most of the genomes we analyzed. However, we did not see the production of this specific sphingolipid. However, we did observe novel sphingolipids, particularly in the lipidome of *P. copri* DSM 18205. Further study would be required to isolate these sphingolipid species and examine their individual effects, as well as the uptake and trafficking of *Prevotella*-derived sphingolipids, to determine whether they are involved in similar metabolic processes as have been observed in *Bacteroides* (12). Although we were able to examine the structures of putative enzymes involved in sphingolipid synthesis using structural prediction software, and we were able to heterologously express PcSPTs to test its function, the development of genetic engineering tools for use in *Prevotella* would be pivotal in identifying and testing additional enzymes involved in these pathways to understand both the role of sphingolipids in *Prevotella* fitness and ultimately to test their impact on host–microbiome interactions. This unique data set of diverse isolates nevertheless provides a fertile ground for examining genetic variation that impacts sphingolipid composition.

*Prevotella* is one of the major genera that has been negatively influenced by rapid industrialization (44, 45). Given the role of *Bacteroides*-derived sphingolipids in maintaining the host’s intestinal homeostasis (11–13), it will be important to investigate whether *Prevotella-*derived sphingolipids provide the same benefits. The exact role of *P. copri* in disease has been debated, and its abundance may be closely tied to specific diets. Given that sphingolipid-producing bacteria have been shown to actively uptake dietary sphingolipids (46), understanding sphingolipid synthesis and utilization could provide an insight into the prominence of this microbe in certain populations. Although sphingolipids produced by oral and respiratory *Prevotella* species have been characterized decades ago (24), the difficulty in cultivating *Prevotella* isolates from the gut and the unequal distribution of research resources in populations where *P. copri* are dominant (47) have precluded its study. This study begins to fill this gap, highlighting differences in sphingolipid production within gut Bacteroidota and even within a set of strains of *Prevotella copri*.

## MATERIALS AND METHODS

### *Prevotella copri* strains and genomes

The type strain used in this study, *Prevotella copri* DSM 18205, was purchased from DSMZ. Its genome, GCF_000157935.1, was downloaded from NCBI RefSeq. The other *P. copri* strains were isolated from a human stool sample collected as part of the Fiji Community Microbiome Project (FijiCOMP) (30). The isolation and characterization of *P. copri* strains were performed using a pipeline established in the lab. The fecal sample was plated on modified Medium 10 (M10, Table S3) plates to obtain single colonies, of which the taxonomies were then identified by Sanger sequencing on 16S rRNA genes. The formula of M10 was adjusted from a previous study (31). The isolates that were confirmed as *P. copri* were prepared into whole-genome sequencing (WGS) libraries following the standard protocol of the NEBNext Ultra II DNA Library Prep Kit for Illumina (NEB) and sequenced on the Illumina NextSeq500 platform using 2 ⨉ 250 bp reads. The paired-end raw reads were processed based on a standard quality control (QC) pipeline established in the lab (48). The paired-end raw reads were trimmed by Trimmomatic (49) and assembled into genomes using SPAdes v3.10.1 (50). Any contigs that were less than 500 bp in length were filtered out. The completeness and quality of assembled genomes were checked with QUAST v4.0 (51) and CheckM v1.0.11 (52) with a contamination cutoff of 5% and completeness cutoff of 95%. The 63 isolate genomes that passed the filtering process plus the *P. copri* DSM 18205 strain were clustered into five clades based on the pangenome analysis of their total gene contents using Anvi’o-7.1 (53) (default settings) and on >400 marker genes using PhyloPhlAn 3.0 (54) (accurate mode). Open-reading frames were predicted by running Prodigal v2.6.3 (55) on obtained genomes. Proteins were annotated from the KEGG (Kyoto Encyclopedia of Genes and Genomes) prokaryotic protein database (56) using DIAMOND v0.9.21 (57) Blastx. Hits with e-values higher than 1e-05 or less than 30% identity to the reference sequences were removed. Sphingolipids were characterized by LC-MS in 39 of these isolates and *P. copri* DSM 18205 (Table S2).

### Bacteria culturing and preservation

The culturing of anerobic bacteria was done inside a vinyl anerobic chamber (Coy Laboratory Products, Inc.) maintained with a gas mix of 3% H_2_, 20% CO_2_, and 77% N_2_. Frozen stocks were first inoculated onto degassed M10 agar plates for 24 hours at 37°C inside the anerobic chamber. They were then subcultured to either BBL Schaedler Broth (BD Biosciences) or M10 plates, depending on the requirements of the particular experiment. *E. coli* BL21 and *E. coli* DH5α were aerobically cultured in the Luria–Bertani medium (LB) at 37°C. Ampicillin was added into the media at 50 µg/mL when selecting for the transformed plasmids.

### Identification and analysis of sphingolipid synthesis genes from *P. copri* genomes

The amino acid sequences of four SPT homologous genes from closely related species, pfus:ADJ77_02970 from *Prevotella fusca*, pdt:Prede_0793 from *Prevotella denticola*, pdn:HMPREF9137_1409 from *Prevotella dentalis*, bth:BT_0870 from *Bacteroides thetaiotaomicron*, and another 15 reviewed SPT proteins from UniProt were searched against all *P. copri* isolates and type strain genomes for potential SPT genes in *P. copri* using BLASTp with a cutoff e-value of 1e-04 and a coverage of 50%. Sequences and source strains of identified SPT gene variants from *P. copri* genomes are listed in Table S1.

The same BLASTp settings and method were used for 3-KDSR, CerS, CerR, and CGT identification as mentioned previously. For CGT, the eight reference sequences used included six reviewed CGT proteins from UniProt and the CGT genes from *B. fragilis* NCTC 9343 (BF9343_3149) and *Zymomonas mobilis* (ZMO1957) (41). For 3-KDSR, the reference sequences contain 21 reviewed 3-KDSR sequences from UniProt and one newly identified sequence from *B. thetaiotaomicron* (BT_0972) (39). For CerS and CerR, sequences from *B. fragilis* (BF9343_4218; BF9343_4240) and *Caulobacter crescentus* (CCNA_01212; CCNA_01222) were used in the homology analysis (26, 37).

Structures for *P. copri* SPTs were predicted using the Evolutionary Scale Modeling (ESMFold) web server (58). For *B. thetaiotaomicron* SPT and the human SPT2-3–KS complex, the AlphaFold (59) Entry Q8A9E5 and PDB entry 7k0k were used respectively.

### *E. coli* BL21*-SPT* LC-MS sample preparation

Two variants of the *P. copri* SPT genes, PcSPT1 and PcSPT2, were cloned onto plasmid pET21b, and the constructed expression vectors were transformed into *E. coli* BL21 for bioactivity verification (primers used are listed in Table S4). *E. coli* BL21 harboring pET21b-PcSPT1 and pET21b-PcSPT2 was cultured in M9 liquid medium with 50 µg/mL ampicillin until the mid-log phase. One milliliter of the liquid cultures was subcultured into 4 mL M9 medium and incubated for 1 hour, after which the following were added to the indicated final concentrations: (1) PAA (10 µM), Cas amino acids (0.2%), and IPTG (100 µM); (2) palmitic acid (PA, 10 µM), casamino acids (0.2%), and IPTG (100 µM). The liquid cultures were incubated overnight at 37°C, after which cells were pelleted by centrifuging at 5,000 g for 10 minutes at 4°C. Cell pellets were washed with sterile PBS twice, and supernatants were carefully removed. The cell pellets were flash-frozen using liquid nitrogen and stored in −80°C until further processing. Samples then underwent processing and lipid extraction for LC-MS (see below).

### Identification and analysis of CGT and 3-KDSR from *P. copri* genomes

The same BLASTp settings and method were used for CGT and 3-KDSR identification as mentioned previously. For CGT, the eight reference sequences used included six reviewed CGT proteins from UniProt, the CGT genes in *B. fragilis* NCTC 9343 (BF9343_3149), and *Zymomonas mobilis* (ZMO1957) (41). For 3-KDSR, the reference sequences contained 21 reviewed 3-KDSR sequences from UniProt.

The BLASTp hits with the highest percent identities were chosen, and the whole sequences of corresponding proteins were pulled out from each genome. The percent identity between reference proteins and predicted proteins from *P. copri* strains was calculated using Clustal Omega MSA tool with default settings (60).

### Preparation of *P. copri* samples for LC-MS

*P. copri* DSM 18205 was streaked onto M10 plates and cultured anerobically for 24 hours. Colonies were collected and suspended in PBS with optical density readings at 600 nm (OD_600_) adjusted to 1.0. One milliliter of the cell suspension was inoculated into 50 mL of pre-warmed degassed Schaedler broth. Once cells reached early-log phase (~6 hours after inoculation), the following reagents were added to the liquid cultures: Group 1: palmitic acid alkyne (PAA, 25 µM) and 50 µL methanol; Group 2: 50 µL ethanol and myriocin (1 µM); Group 3: PAA (25 µM) and myriocin (1 µM); Group 4: 50 µL ethanol and methanol. Adding myriocin at the early-log phase instead of prior to subculturing helps reduce uneven growth of bacteria in different groups due to the inhibition of growth by myriocin. Cells were then cultured anerobically at 37°C for 24 hours and then pelleted by centrifuging at 6,000 g for 10 minutes at 4°C. Supernatants were discarded, and the cells were washed with 10 mL sterile PBS and spun down using the same centrifuge settings. The pellets were suspended in 1 mL PBS and transferred into a 1.5-mL centrifuge tube. Cells were finally pelleted by centrifuging at 6,000 g for 5 minutes at 4°C. Supernatants were carefully removed, and the cell pellets were flash-frozen with liquid nitrogen and stored at −80°C before processing for liquid high-resolution LC-MS.

### Sample preparation of *P. copri* isolates

To prepare the samples for mass spectrometry, 39 *P*. *copri* strains and *P. copri* DSM 18205 were inoculated into 10 mL of pre-warmed degassed Schaedler broth. At the early-log phase, PAA (25 µM) was added to the liquid cultures, and the same amount of ethanol was added to an extra tube of *P. copri* DSM 18205 as a negative control. The tubes were cultured anerobically for 24 hours, and the cells were collected following the same procedures mentioned previously. Reagents and stock solutions used are listed in Table S5.

### Sample processing and lipid extraction for LC-MS

Bacterial cell pellets were frozen in liquid nitrogen and lyophilized to dryness. One milliliter of HPLC-grade methanol was added to the dried material, and the mixture was sonicated for 3 minutes (on/off pulse cycles of 2 seconds on, 2 seconds off, at power 100%) using a Qsonica Ultrasonic Processor (Model Q700) with a water bath cup horn adapter (Model 431C2), with water bath flow to maintain room temperature. Samples were then moved to an end-over-end rotator, and extractions proceeded for 12 hours. Samples were then centrifuged at 18,000 × *g* for 30 minutes at 4°C. The supernatant was transferred to a fresh centrifuge tube, and the solvent was dried with a Thermo Scientific Savant SpeedVac SPD130DLX. The dried material was resuspended in 200 µL HPLC-grade methanol, briefly sonicated, and centrifuged as before. The concentrated extract was transferred to an HPLC vial with a 300-µL glass insert and stored at 4°C until further analysis.

### LC-MS methods for lipid analysis of *P. copri* isolates

Low-resolution LC−MS analysis was performed on a ThermoFisher Scientific Vanquish Horizon UHPLC System coupled with a ThermoFisher Scientific TSQ Quantis Triple–Quadrupole mass spectrometer equipped with an HESI ion source. One microliter of the extract was injected and separated on a mobile phase gradient with an Agilent Technologies InfinityLab Poroshell 120 EC-C18 column (50 mm × 2.1 mm, particle size 2.7 µm, part number: 699775–902) maintained at 50°C.

#### LC-MS positive mode analysis

Mobile phase A was 78.6% water, 20% acetonitrile, and 0.4% formic acid. Mobile phase B was 47.8% methanol, 47.8% acetonitrile, 4% chloroform, and 0.4% formic acid. A/B gradient was started at 10% B for 1 minute after injection and increased linearly to 100% B at 9 minutes and held at 100% B for 10 min, using a flow rate 0.6 mL/min. Full Scan Q1 mass spectrometer parameters: spray voltage, 2.5 kV for negative mode; ion transfer tube temperature, 350°C; vaporizer temperature, 350°C; sheath, auxiliary, and spare gas, 60, 15, and 2, respectively. Tandem mass spectrum analysis was carried out with Product Ion Scan mode utilizing the same parameters (see above) with the following additions: collision energy: 30 V; CID gas 1.5 mTorr.

#### LC-MS negative mode analysis

Mobile phase A was 94.9% water, 5% methanol, and 0.1% formic acid (vol/vol) with 10 mM ammonium acetate. Mobile phase B was 99.9% methanol and 0.1% formic acid (vol/vol). A/B gradient was started at 15% B for 1 minute after injection and increased linearly to 100% B at 22 minutes and held at 100% B for 5 minutes, using a flow rate 0.6 mL/min. Full Scan Q1 mass spectrometer parameters: spray voltage, 2.0 kV for negative mode; ion transfer tube temperature, 350°C; vaporizer temperature, 350°C; sheath, auxiliary, and spare gas, 60, 15, and 2, respectively. Tandem mass spectrum analysis was carried out with Product Ion Scan mode with the following additions: collision energy: 30 V; CID gas 1.5 mTorr.

High-resolution LC-MS analysis was performed on a Thermo Fisher Scientific Vanquish Horizon UHPLC System coupled with a Thermo Q Exactive HF hybrid quadrupole-orbitrap high-resolution mass spectrometer equipped with an HESI ion source. One microliter of the extract was injected and separated using a water–acetonitrile gradient on a Kinetex EVO C18 column (150 mm 3 2.1 mm, particle size 1.7 mm, part number: 00F-4726-AN) maintained at 40°C. Solvent A: 0.1% formic acid in water; Solvent B: 0.1% formic acid in acetonitrile. A/B gradient was started at 10% B for 3 minutes after injection and increased linearly to 100% B at 17 minutes and held at 100% B for 10 min, using a flow rate of 0.5 mL/min. Mass spectrometer parameters: spray voltage, 3.5 kV for positive mode and 3.0 kV for negative mode; capillary temperature, 380°C; prober heater temperature, 400°C; sheath, auxiliary, and spare gas, 60, 20, and 1, respectively; S-lens RF level 50, resolution 240,000 at m/z 200, AGC target 3 × 10^6^. Each sample was analyzed in positive and negative modes, with m/z ranging from 100 to 1,200.

### Untargeted metabolomic analysis

RAW files generated from high-resolution LC-MS acquisitions were converted to mzXML files utilizing MSconvertGUI software (proteowizard.sourceforge.net). Differential metabolic features were determined by subjecting mzXML files to Metaboseek Software version 0.9.6 (metaboseek.com) (Helf et al., 2022) utilizing the XCMS package with all values set to default parameters which are as follows: peak detection: ppm 4, peak width 320, snthresh 3, prefilter 3100, fitgauss FALSE, integrate 1, firstBaselineCheck TRUE, noise 0, mzCenterFun wMean, mzdiff −0.005, and workers 4; peak filling: METABOSEEK, ppm_m 5, rtw 5, rtrange TRUE, and areaMode FALSE; feature grouping: minfrac 0.2, bw 2, mzwid 0.002, max 500, minsamp 1, and usegroups FALSE; CAMERA and RT correction were not used. Differential molecular features were sorted using the minFoldOverCtrl, minInt, and Fast_Peak_Quality filters. Differential features were subjected to manual curation to remove adducts, isotopes, and false positives. The curated list of molecular features was assigned molecular formulas.

### Isotope-labeled serine experiment

*P. copri* DSM 18205 was subcultured into 10 mL of warm degassed Schaedler broth from the M10 plate and anerobically cultured at 37°C. Once the bacteria reached the early-log phase, either PAA (25 µM) or ethanol and/or L-Serine (10 mM) were added. After 24 hours, cells were pelleted using a centrifuge at 6,000 g for 5 minutes at 4°C, washed with 1 mL sterile PBS, and spun down again. Cell pellets were flash-frozen using dry ice and stored at −80°C.

## Data Availability

The whole-genome sequencing data of *Prevotella copri* isolates included in this paper are available under NCBI BioProject PRJNA217052. The accession numbers of all corresponding SRAs and BioSamples are listed in Table S6.

## SUPPLEMENTAL MATERIAL

Supplemental figure mbio.02409-23-s0001.tif Figures S1.

Supplemental tables mbio.02409-23-s0002.docx Table S1 to S6.

ASM does not own the copyrights to Supplemental Material that may be linked to, or accessed through, an article. The authors have granted ASM a non-exclusive, world-wide license to publish the Supplemental Material files. Please contact the corresponding author directly for reuse.
