# Supplementary figures and images for: Prevotella copri variants among a single host diverge in sphingolipid production
Source: mBio. 2024 Jan 18;15(2):e02409-23. doi: 10.1128/mbio.02409-23 (PMC10865984; doi:10.1128/mbio.02409-23)

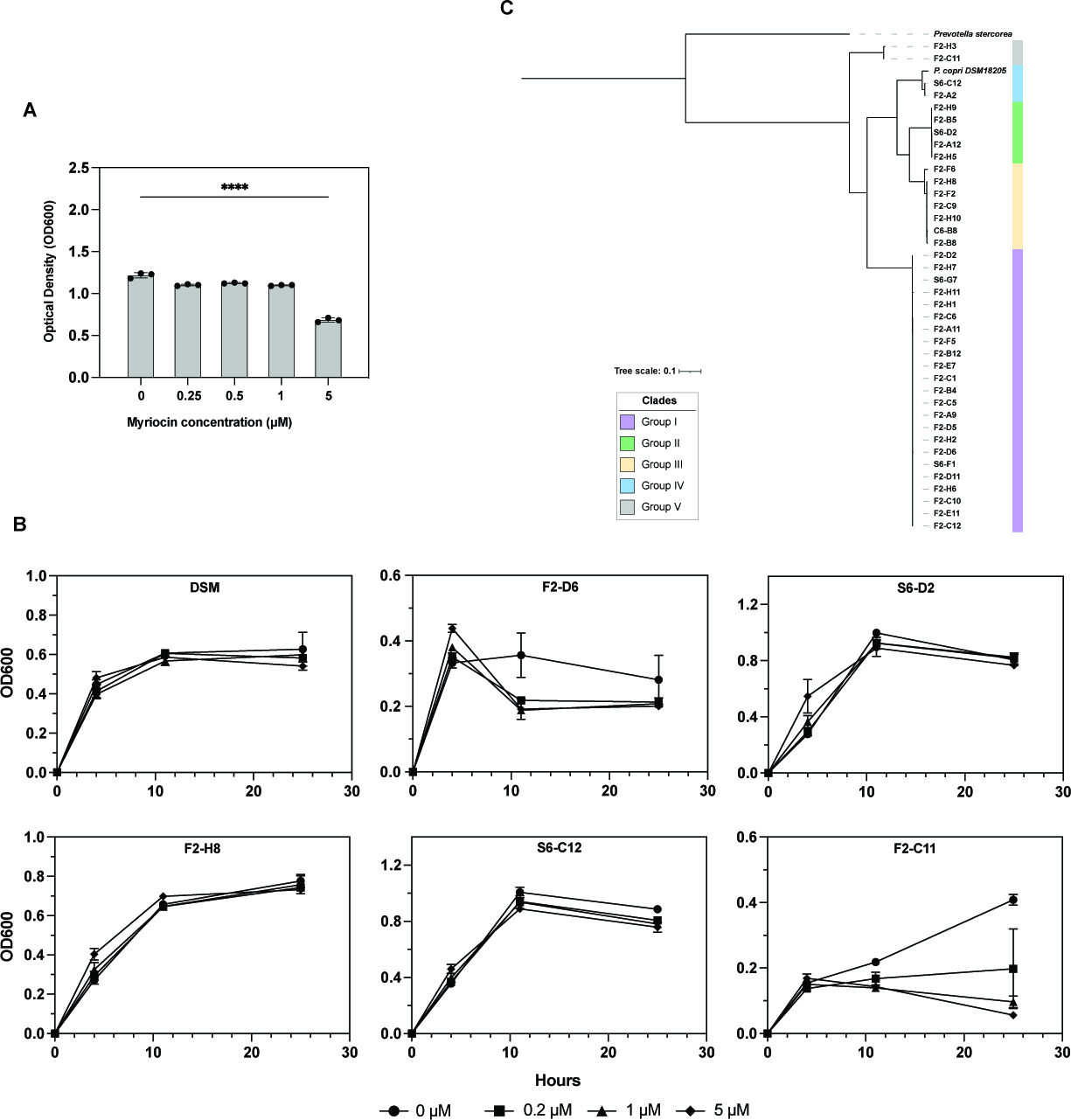

Supplement: Supplemental figure — Figures S1. [file mbio.02409-23-s0001.tif]
