# Supplementary material for: Prevotella copri variants among a single host diverge in sphingolipid production
Source: mBio. 2024 Jan 18;15(2):e02409-23. doi: 10.1128/mbio.02409-23 (PMC10865984; doi:10.1128/mbio.02409-23)
Supplement: Supplemental tables — Table S1 to S6. [file mbio.02409-23-s0002.docx]

***Prevotella copri* variants among a single host diverge in sphingolipid production**

Xieyue Xiao^1#^, Henry H. Le^2#^, Min-Ting Lee^2^, Daniel Lamm^1^, Elizabeth L. Johnson^2,3^*, Ilana L. Brito^1^*

^1^Meinig School of Biomedical Engineering, Cornell University, Ithaca, NY, USA.

^2^Division of Nutritional Sciences, Cornell University, Ithaca, NY, USA.

^3^Howard Hughes Medical Institute, Chevy Chase, MD, USA

^#^ Contributed equally.

* Please send all correspondences to [ibrito@cornell.edu](mailto:ibrito@cornell.edu) and [elj54@cornell.edu](mailto:elj54@cornell.edu).

**Supplement information**

**Supplemental Figure 1. Experimental considerations for sphingolipid characterization.**

(A) The stationary phase optical density at 600 nm (OD_600_) of *P. copri* DSM 18205 with gradients of myriocin added prior to subculturing (multiple t-test with Bonferroni correction, ****: p ≤ 0.0001). (B) Growth curves of 5 select *P. copri* isolates, representing each of the five clades, and *P. copri* DSM 18205 with and without the addition of myriocin at early log phase. Error bars indicating mean ± SD (n=3). (C) The 40 *P. copri* isolates used in this study harbor genomic variations and cover all five distinct clades of all *P. copri* isolates. Clustering was done by PhyloPhlAn 3.0.

**Table S1. Potential SPT gene variants identified from *P. copri* genomes.**

|  | **Sequence** | **Source strain** |
| --- | --- | --- |
| **PcSPT1** | ATGGGACAATTACAAGAAAGATACAAGAATTATCGTGAACCTCAGAAGTATATGGCTGCCGGTGTGTATCCATATTTCCGCGAGATCACAAGTAAGCAGATGACAGAGGTAACCGACATCGATGGTCATAAAATTCTGATGTTCGGTTCTAATGCTTATCAGGGTTTGACTAACGACCAGCGTGTTATTGATGCAGCAAAGGCTGCGCTCGACAAGTATGGTTCTGGTTGTGCAGGAAGTCGTTTCCTCAATGGTACGCTCGATTTGCACGTGCAGCTCGAAAAGGAGCTTGCTGAGTTTATGGGCAAGGATGAAACTTTGTGTTTCTCTACAGGATTCTCTGTAAACCAGGGTGTCTTGGCTTGCGTAGTAGGTCGTAACGACTATATTATCTGCGATGACCGCGACCACGCAAGTATCGTAGATGGCCGCCGCCTCTCATTCGCTACCCAGCTCCACTACAAGCACAACGATATGGAAGATTTGGAACGCGTGCTCTCTAAGCTCCCTCAGGAGGCTATCAAGCTCATCGTTGTTGACGGTGTGTTCTCTATGGAGGGCGACTTGGCTAACTTGCCTGAGATTGTAAAGCTCAAGCATAAGTACAACTGCTCTATCATGGTAGACGAGGCTCATGGTCTTGGCGTATTCGGCAAGCAGGGTCGTGGTGTATGCGACCACTTCGGTCTGACCGACGAGGTAGACCTCATCATGGGTACATTCTCTAAGAGTCTGGCTTCTATCGGCGGTTTCATCGCATCAGACAAGGATACTATCAACTTCCTCCGTCACAACTGCCGTACTTACATCTTCAGCGCCTCTAACACTCCAGCTGCTACAGCAGCAGCTCTCGAGGCTCTCCACATCATTCAGAATGAGCCAGAGCGTTTCGAGAATCTCTGGGATGTAACCAACTATGCCTTGAAGCGTTTCCGCGAGGAAGGTTTCGAGATTGGCGAGACAGAGAGTCCTATCATTCCTCTCTACGTACGCGATGCTGAGAAGACATTCGTTGTTACCAAGATGGCTTACGATGCAGGTGTGTTCATCAACCCTGTTATTCCTCCAGCTTGTGCTCCTCAGGATACATTGGTACGTTTCGCACTCATGGCTACTCATACAAGAGAGCAGGTTGAGCAGGCTGTCCAGATCTTGAAGAAGATTTTCGTAGAAGAGGGAATCATCAAGTAA | F2-A2, S6-C12 |
| **PcSPT2** | ATGGGACAATTACAAGAAAGATACAAGAATTATCGTGAACCACAGAAGTACATGGCTGCCGGCGTGTATCCTTACTTCCGTGAAATCACAAGTAAGCAGATGACAGAGGTAACCGATATTGATGGTCATAAGATTTTGATGTTCGGTTCTAATGCTTATCAGGGTTTAACCAACGACCAGCGCGTAATCGATGCTGCCAAGGCTGCGCTCGACAAGTATGGTTCTGGTTGTGCAGGAAGCCGTTTCCTCAATGGTACCCTTGACTTGCACGTTCAGTTGGAAAAGGAACTGGCTGATTTTATTGGTAAGGACGAGACACTTTGCTTCTCTACAGGTTTCTCTGTAAACCAAGGTGTTTTGGCTTGCGTTGTAGGTCGTAACGACTATATCATCTGCGACGATCGTGACCACGCTTCTATCGTAGATGGTCGCCGTCTCTCATTCGCTACTCAGCTTCACTACAAGCACAACGACATGGAAGACTTGGAGCGCGTGCTCGCTAAGCTCCCTCAGGAGGCTATCAAGCTGATCGTTGTTGACGGTGTGTTCTCTATGGAGGGTGACCTCGCCAACTTGCCTGAGATCGTGAAGTTGAAGCACAAGTACAACTGCTCTATCATGGTCGATGAGGCTCATGGCTTGGGCGTATTTGGTAAGCAGGGTCGTGGCGTTTGCGACCATTTCGGCTTGACCGATGAGGTGGATCTCATCATGGGTACATTCTCTAAGAGTTTAGCATCTATCGGTGGTTTCATTGCATCTGACAAGGATACGATCAACTTCCTCCGTCATAACTGCCGCACTTATATCTTCAGTGCTTCCAACACTCCAGCTGCTACTGCTGCCGCACTTGAGGCATTGCACATCATCGAGAATGAGCCAGAGCGTTTCGAGAATCTTTGGGATGTTACCAACTATGCTTTGAAGCGTTTCCGTGAGGAAGGTTTCGAGATTGGTGAGACAGAGAGCCCTATCATTCCACTTTATGTGCGTGATGCAGAGAAGACTTTCCTTGTAACCAAGAAGGCTTACGATCGTGGTGTGTTCATCAACCCTGTGATTCCTCCAGCATGTGCTCCTCAGGACACATTGGTACGTTTCGCTTTGATGGCTACTCATACCAAGGAGCAAGTAGAGCGGGCTGTTGTTATCTTGAAGCAGATTTTCGTAGAGGAAGGTATCATCAAGTAA | F2-A11, F2-A9, F2-B12, F2-B4, F2-C1, F2-C10, F2-C12, F2-C5, F2-D11, F2-D2, F2-D5, F2-D6, F2-E11, F2-E7, F2-F5, F2-H1, F2-H11, F2-H2, F2-H6, F2-H7, C6-A1, C6-A2, C6-B3, C6-D1, C6-H5, C6-H7, S6-C1, S6-C2, S6-D10, S6-D1, S6-D7, S6-F1, S6-G6, S6-G7, S6-G8, S6-H5 |
| **PcSPT3** | ATGGGACAATTACAAGAAAGATACAAGAATTATCGTGTTCCTCAGAAGTATATGGATGCCGGTGTGTATCCATACTTCCGCGAGATCACAAGTAAGCAGATGACAGAAGTTACCGACATCGACGGACACCACATCCTGATGTTCGGTTCTAATGCTTATCAGGGTTTGACAAACGACCAGCGTGTCATCGATGCAGCCAAGGCTGCTCTTGACAAGTATGGCTCAGGCTGTGCGGGTAGTCGTTTCCTCAATGGTACCCTCGATTTACATGTACAGCTCGAGAAGGAGCTTGCAGAGTTTATGGGTAAGGACGAGACATTGTGCTTCTCTACTGGTTTCTCTGTGAACCAGGGTGTGTTGGCATGTGTTGTGGGTCGCGGTGATTATATCATCTGCGACGACCGTGACCACGCCAGTATCGTAGATGGCCGCCGTCTCTCATTCGCTACCCAGCTTCACTACAAGCACAACGACATGGAAGATTTGGAGCGCGTGCTCTCTAAGCTTCCTGAGGATGCTGTCAAGCTCATCGTGGTTGATGGCGTATTCTCTATGGAGGGTGATTTGGCCAACTTGCCTGAGATTGTAAAGCTCAAGCATAAGTACAACTGCTCTATCATGGTGGATGAGGCTCATGGTCTCGGTGTATTCGGTAAGCAGGGACGTGGTGTCTGCGACCACTTCGGCTTGACAGATGAGGTAGACCTTATCATGGGTACATTCTCTAAGAGTTTGGCTTCTATCGGTGGTTTCATCGCATCAGACAAGGATACTATCAACTTCCTCCGTCACAACTGCCGTACTTACATCTTTAGTGCATCCAATACTCCAGCAGCTACCGCTGCTGCTCTTGAGGCACTCCACATCATTCAGAAAGAGCCAGAGCGTTTCAAGAATCTGTGGGATGTAACTAACTATGCCTTGAAGCGTTTCCGCGAGGAGGGTTTTGAGATTGGTGAGACCGAGAGTCCTATCATTCCTCTCTACGTTCGCGATGCTGAGAAGACATTCGTTGTTACTAAGTTGGCTTACGATGCGGGTGTATTCATCAATCCGGTTATTCCTCCAGCATGTGCTCCACAGGATACATTGGTACGTTTTGCCCTCATGGCAACTCATACCAAGGATCAGGTAGAGAAGGCTGTTCAGATTTTGAAGAAGATTTTCGTAGAGGAAGGAATCATCAAGGCGTAA | F2-A12, F2-B5, F2-H5, F2-H9, C6-B1, S6-C3, S6-C8, S6-D2, S6-E10, S6-E12, S6-E7, S6-F11 |
| **PcSPT4** | ATGGGACAATTACAAGAAAAATACAAGAATTATCGTGAACCTCAGAAGTATATGGCTGCCGGTGTGTACCCATACTTCCGTGAGATCACCAGTAAGCAGATGACGGAAGTTACCGACATCGATGGACATAAGATTTTGATGTTCGGTTCTAATGCTTATCAGGGTTTGACCAACGACCAGCGTGTTATCGATGCAGCCAAGGCTGCTCTCGACAAGTATGGTTCTGGTTGTGCAGGAAGCCGTTTCCTCAATGGAACCCTCGACCTTCACGTACAGCTCGAGAAGGAGTTGGCGGAGTTCATGCACAAGGATGAGTCCCTGTGCTTCTCTACAGGTTTCTCTGTCAACCAGGGCGTGTTGGCTTGTGTGGTAGGCCGCAATGACTATATCATCTGCGACGACCGCGACCACGCCAGTATCGTAGACGGCCGCCGTCTTTCGTTTGCAACCCAGCTGCACTATAAGCACAATGATATGGAGGACCTTGAGCGCGTACTCAAGACCTTGCCTCATGAGGCTATCAAGCTGATCGTTGTCGATGGCGTCTTCTCTATGGAGGGCGACCTGGCCAACTTGCCAGAGATTGTCAAGTTGAAGCACAAGTACAACTGCTCCATCATGGTGGACGAGGCTCATGGCCTGGGTGTGTTTGGCAAGCAGGGACGTGGCGTGTGCGATCACTTTGGTCTTACCGACGAGGTCGACCTCATCATGGGTACATTCTCCAAGAGTCTGGCGTCTATCGGTGGTTTCATCGCATCAGACAAGGACACCATCAACTTCCTGCGTCATTCTTGCCGTACTTATATCTTCAGTGCATCCAACACTCCAGCAGCCACAGCAGCAGCCTTGGAGGCTCTTCACATCATTCAGAACGAGCCAGAGCGCTTCGAGCAGTTGTGGGATGTTACCAACTACGCCCTGAAGCGTTTCCGTGAGGAAGGTTTCGAGATTGGCGAGACAGAGAGTCCTATCATTCCTCTCTATGTGCGCGATGCTATGAAGACTTTCGAGGTAACCAAGAAGGCTTACGACCGTGGCGTATTCATCAACCCAGTCATCCCTCCAGCATGTGCTCCTCAGGACACATTGGTACGTTTCGCCCTCATGGCTACTCATACCAAGGAGCAGGTAGAACGCGGTGTGAAAATTCTGAAGGAGGTCTTCGTAGAGGAAGGCGTCATCAAGTAA | F2-C11, F2-H3, S6-F9 |
| **PcSPT5** | ATGGGACAATTACAAGAAAGATACAAGAATTATCGTGTACCTCAGAAGTATATGGAAGCCGGTGTGTATCCATACTTCCGTGAGATTACAAGTAAGCAGATGACAGAGGTAACTGACATCGACGGTCACAAGATCCTGATGTTCGGTTCTAATGCTTACCAGGGTTTGACAAATGATCAGCGTGTTATCGATGCAGCTAAGGCTGCTCTTGACAAGTACGGCTCAGGCTGTGCTGGTAGCCGCTTCCTCAATGGTACACTCGATTTGCATGTGCAGCTCGAAAAAGAGCTTGCTGAGTTTATGGGCAAGGATGAGACATTGTGCTTCTCTACTGGTTTCTCTGTAAACCAGGGCGTGTTGGCATGTGTTGTAGGCCGCGGTGATTATATCATCTGTGACGACCGTGATCATGCCAGTATCGTAGATGGCCGTCGTCTTTCATTCGCTACCCAGCTTCACTACAAGCACAACGATATGGAAGATTTGGAGCGCGTGCTCTCTAAGCTTCCTGAGGATGCAGTCAAGCTCATCGTTGTGGATGGCGTGTTCTCTATGGAGGGCGATTTGGCTAACTTGCCAGAAATCGTGAAGTTGAAGCATAAGTACAACTGCTCTATCATGGTAGATGAGGCTCATGGCCTTGGCGTGTTTGGTAAGCAGGGACGTGGTGTCTGCGATCATTTCGGTCTGACCGATGAGGTAGATCTCATCATGGGTACCTTCTCTAAGAGTCTGGCATCTATCGGTGGTTTCATCGCATCAGATAAGGATACCATCAATTTCCTCCGTCACAACTGCCGTACTTACATCTTCAGTGCATCTAACACTCCAGCTGCAACTGCTGCAGCTCTCGAGGCTCTCCATATCATCCAGAAGGAGCCTGAGCGTTTTGAGAATCTTTGGGATGTTACCAACTATGCTTTGAAGCGTTTCCGTGAGGAAGGTTTCGAGATTGGTGAGACTGAGAGTCCTATCATTCCTCTCTACGTTCGTGATGCAGAGAAGACATTCGTTGTTACCAAGCTGGCTTATGATGCAGGTGTATTCATCAATCCTGTTATCCCACCAGCATGTGCTCCTCAGGACACATTGGTGCGTTTTGCTCTTATGGCTACTCATACAAGAGAGCAGGTAGAGAAGGCTGTTGAGATTTTGAAGAAGATTTTCGTAGAGGAAGGCATCATCAAGGCGTAA | F2-B8, F2-C9, F2-F2, F2-H10, F2-H8, C6-B8, C6-D3 |
| **PcSPT6** | ATGGGACAATTACAAGAAAGATACAAGAATTATCGTGTACCTCAGAAGTATATGGAAGCCGGTGTGTATCCATACTTCCGTGAGATCACAAGTAAGCAGATGACAGAGGTAACTGACATCGATGGTCATAAGATCCTGATGTTCGGTTCTAATGCTTACCAGGGTTTGACTAATGATCAGCGAGTTATCGATGCAGCTAAGGCTGCTCTCGACAAGTACGGCTCTGGCTGTGCTGGTAGCCGCTTCCTCAATGGTACACTCGATTTGCATGTGCAGCTCGAAAAAGAGCTTGCTGAGTTTATGGGCAAGGATGAGACATTGTGCTTCTCTACTGGTTTCTCTGTAAACCAGGGCGTGTTGGCATGTGTTGTAGGCCGCGGTGATTATATCATCTGTGACGACCGTGATCATGCCAGTATCGTAGATGGCCGTCGTCTTTCATTCGCTACCCAGCTTCACTACAAGCACAACGATATGGAAGATTTGGAGCGCGTGCTCTCTAAGCTTCCTGAGGATGCAGTCAAGCTCATCGTTGTGGATGGCGTGTTCTCTATGGAGGGCGATTTGGCTAACTTGCCAGAAATCGTGAAGTTGAAGCATAAGTACAATTGCTCTATCATGGTAGATGAGGCTCATGGCCTTGGCGTGTTTGGTAAGCAGGGACGTGGTGTCTGCGATCATTTCGGCTTGACAGATGAGGTGGATCTTATCATGGGTACCTTCTCTAAGAGTCTGGCATCTATCGGTGGTTTCATCGCTTCTGATAAGGATACTATCAATTTCCTCCGTCACAACTGCCGTACTTACATCTTCAGTGCATCTAACACTCCAGCTGCAACTGCTGCTGCTCTCGAGGCTCTCCATATCATCCAGAAGGAGCCAGAGCGTTTTGAGAATCTTTGGGATGTTACCAACTATGCTTTGAAGCGTTTCCGTGAGGAAGGTTTCGAGATTGGTGAGACTGAGAGTCCTATCATTCCTCTCTACGTTCGTGATGCAGAGAAGACATTCGTTGTTACCAAGCTGGCTTATGATGCAGGTGTGTTCATCAATCCTGTTATCCCACCAGCATGTGCTCCTCAGGACACATTGGTGCGTTTTGCTCTTATGGCTACTCATACAAGAGAGCAGGTAGAGAAGGCTGTTGAGATTTTGAAGAAGATTTTCGTAGAGGAAGGCATCATCAAGGCGTAA | F2-F6 |
| **PcSPT7** | ATGGGACAATTACAAGAAAGATACAAGAATTATCGTGAACCACAGAAGTATATGGCTGCCGGTGTGTATCCATATTTCCGTGAAATCACAAGTAAGCAGATGACAGAAGTTACCGACATTGACGGTCACAAGATTTTGATGTTCGGTTCTAATGCTTATCAGGGTTTAACCAACGACCAGCGCGTTATCGATGCAGCTAAGGCTGCGCTCGACAAGTACGGTTCTGGCTGTGCAGGAAGCCGCTTCCTCAATGGTACCCTCGATTTACATGTGCAGCTCGAGAAAGAGCTCGCTGAGTTTATGGGTAAGGACGAGACATTGTGCTTCTCTACTGGTTTCTCTGTAAACCAGGGTGTGTTGGCATGTGTTGTTGGTCGTAACGACTATATCATCTGTGATGATCGCGACCATGCTTCTATCGTAGATGGCCGTCGTCTCTCTTTCGCTACCCAGCTTCACTACAAGCACAACGACATGGAAGATTTGGAGCGTGTGCTCTCTAAGCTTCCTGAGGATGCTATCAAGCTGATCGTGGTAGACGGTGTGTTCTCTATGGAAGGCGATTTGGCTAACTTGCCTGAGATCGTAAAGCTCAAGCATAAGTACAACTGCTCTATCATGGTTGACGAGGCTCATGGTCTTGGTGTATTCGGCAAGCAGGGACGTGGTGTTTGCGACCACTTTGGCTTGACCGATGAGGTAGACCTCATCATGGGTACCTTCTCAAAGAGTCTGGCATCTATCGGTGGTTTCATCGCATCAGACAAGGATACCATCAACTTCCTCCGTCACAACTGCCGTACCTACATCTTCAGTGCATCCAATACTCCAGCAGCTACAGCAGCAGCGCTCGAGGCACTCCACATCATTCAGAATGAGCCAGAGCGTTTCGAGAATCTCTGGAATGTAACCCACTATGCATTGAAGCGTTTCCGCGAGGAAGGTTTCGAGATTGGCGAGACAGAGAGTCCTATCATTCCTCTCTACGTACGCGATGCTGAGAAGACATTCGTTGTTACCAAGATGGCTTACGATGCAGGTGTGTTCATCAACCCTGTTATTCCTCCAGCTTGTGCTCCTCAGGATACATTGGTACGTTTCGCACTCATGGCTACTCATACAAGAGAGCAGGTTGAGCAGGCTGTACAGATCTTGAAGAAGATCTTCGTAGAGGAAGGCATCATCAAGTAA | C6-F5 |
| **PcSPT-dsm** | ATGGGACAATTACAAGAAAGATACAAGAATTATCGTGAACCTCAGAAGTATATGGCTGCCGGTGTGTATCCATATTTCCGCGAGATCACAAGTAAGCAGATGACAGAAGTAACCGACATCGATGGTCATAAAATCCTGATGTTCGGTTCTAATGCTTATCAGGGTTTGACTAACGACCAGCGTGTTATTGATGCAGCAAAGGCTGCGCTCGACAAGTATGGTTCTGGTTGTGCAGGAAGCCGTTTCCTCAATGGTACGCTCGATTTGCACGTGCAGCTCGAAAAGGAGCTTGCTGAGTTTATGGGCAAGGATGAAACTTTGTGTTTCTCTACAGGATTCTCTGTAAACCAGGGTGTTTTGGCTTGCGTAGTAGGTCGTAACGACTATATTATCTGCGATGACCGCGACCACGCAAGTATCGTAGATGGCCGCCGCCTCTCATTCGCTACCCAGCTTCACTACAAGCACAACGATATGGAAGATTTGGAGCGCGTGCTCTCTAAACTCCCTGAGGAGGCTATCAAGCTCATCGTTGTTGACGGTGTGTTCTCTATGGAGGGCGACCTGGCTAACTTGCCTGAAATCGTTAAGCTCAAGCATAAGTACAACTGCTCTATCATGGTAGACGAGGCTCATGGTCTTGGCGTATTCGGCAAGCAGGGTCGTGGTGTATGCGACCACTTCGGTCTGACCGATGAGGTAGACCTCATCATGGGTACATTCTCTAAGAGTCTGGCTTCTATCGGCGGTTTCATCGCATCAGACAAGGATACCATCAACTTCCTCCGTCACAACTGCCGTACTTACATCTTCAGCGCCTCTAACACTCCAGCTGCCACAGCAGCAGCTCTCGAGGCACTCCACATCATTCAGAATGAGCCAGAGCGTTTCGAGAATCTCTGGGATGTAACCAACTATGCCTTGAAGCGTTTCCGCGAGGAAGGTTTCGAGATTGGCGAGACAGAGAGTCCTATCATTCCTCTCTACGTACGCGATGCTGAGAAGACATTCGTTGTTACCAAGATGGCTTACGATGCAGGTGTGTTCATCAACCCTGTTATTCCTCCAGCTTGTGCTCCTCAGGATACATTGGTACGTTTCGCACTCATGGCTACTCATACAAGAGAGCAGGTTGAGCAGGCTGTCCAGATCTTGAAGAAGATTTTCGTAGAAGAGGGAATCATCAAGTAA | *P. copri DSM 18205* |

**Table S2. information on *P. copri* strains used in lipidomic studies**

| **Strain** | **Genomic group** |
| --- | --- |
| S6-F1 | I |
| S6-G7 | I |
| F2-H7 | I |
| F2-H6 | I |
| F2-H2 | I |
| F2-H11 | I |
| F2-H1 | I |
| F2-F5 | I |
| F2-E7 | I |
| F2-E11 | I |
| F2-D6 | I |
| F2-D5 | I |
| F2-D2 | I |
| F2-D11 | I |
| F2-C6 | I |
| F2-C5 | I |
| F2-C12 | I |
| F2-C10 | I |
| F2-C1 | I |
| F2-B4 | I |
| F2-B12 | I |
| F2-A9 | I |
| F2-A11 | I |
| S6-D2 | II |
| F2-H9 | II |
| F2-H5 | II |
| F2-B5 | II |
| F2-A12 | II |
| C6-B8 | III |
| F2-H8 | III |
| F2-H10 | III |
| F2-F2 | III |
| F2-C9 | III |
| F2-B8 | III |
| F2-F6 | III |
| F2-A2 | IV |
| *P. copri DSM18205* | IV |
| S6-C12 | IV |
| F2-H3 | V |
| F2-C11 | V |

**Table S3. Formula and preparation of Medium 10 agar**

| **Components** | **Amount in 1L medium** |
| --- | --- |
| Water | 960 mL |
| Glucose | 0.5 g |
| Cellobiose | 0.5 g |
| Soluble starch | 0.5 g |
| Minerals stock | 20 mL |
| L-cysteine-HCl | 0.5 g |
| Resazurin | 2 mL |
| Na_2_CO_3_ | 4 g |
| Trypticase peptone | 2 g |
| Yeast extract | 0.5 g |
| Volatile fatty acid mix | 3.1 mL |
| Hemin | 20 mL |
| Agar | 20 g |
| K_2_HPO_4_ (after autoclave) | 1 mL |

**Preparation**

Dissolve all the components except K_2_HPO_4_ in 960 mL distilled water and autoclave at 121°C for 15 min. Cool the medium down to 50-60°C, add in 1 mL K_2_HPO_4_ and mix well. Pour the medium into plates. After the plates are solidified, transfer into anaerobic chamber to degas overnight.

**Stock solutions**

Minerals solution (1L):

Minerals are prepared into a stock solution and store at room temperature. Mix well before use.

|  | Amount in 1L solution (g) |
| --- | --- |
| KH_2_PO_4_ | 0.177 |
| NaCl | 0.044 |
| (NH)_2_SO_4_ | 0.449 |
| K_2_HPO_4_ (separately) | 0.296 |
| CaCl_2_ | 0.046 |
| MgSO_4_•7H_2_O | 0.094 |

Fatty acids mix:

|  | Volume in the stock (37 mL) |
| --- | --- |
| Acetic acid | 17 mL |
| Propionic acid | 6 mL |
| Butyric acid | 4 mL |
| Isobutyric acid | 1 mL |
| n-valeric acid | 1 mL |
| Isovaleric acid | 1 mL |
| DL-alpha-methylbutyric acid | 1 mL |

Hemin stock solution (0.5 mg/mL):

Dissolve 50 mg hemin in 1 ml 1 N NaOH; make up to 100 ml with distilled water. Store refrigerated and avoid light.

K_2_HPO_4_ stock solution (296.106 g/L):

Dissolve K_2_HPO_4_ in distilled water, filter through 0.22 μm filter.

Resazurin stock solution (0.05%):

Dissolve resazurin in distilled water. Store refrigerated and avoid light.

**Table S4. Primer sequences**

| **Primer** | **Sequence** | **Usage** |
| --- | --- | --- |
| cand1-F | GGAGATATACATATGGGACAATTACAAGAAAGATACAAGAATTATCGTGAACCTCAGAAG | Amplification of PcSPT1 from *P. copri* genome and assemble it to the vector. |
| cand1-R | TGCTCGAGTGCGGCCGCCTTGATGATTCCCTCTTCTACGAAAATCTTCTTCAAGATCTGG |  |
| cand2-F | GGAGATATACATATGGGACAATTACAAGAAAGATACAAGAATTATCGTGAACCACAGAAG | Amplification of PcSPT2 from *P. copri* genome and assemble it to the vector. |
| cand2-R | GGTGGTGCTCGAGCTTGATGATACCTTCCTCTACGAAAATCTGCTTCAAGATAACAACAG |  |
| pET21b-check-fw | CCGCGAAATTAATACGACTC | Verification of transformation in *E. coli*. |
| pET21b-check-rev | ATCCGGATATAGTTCCTCC |  |

**Table S5. Reagent stocks preparation**

|  | **Stock concentration** | **Solvent** | **Preparation** |
| --- | --- | --- | --- |
| **PA** | 25 mM | Ethanol | Dissolve Palmitic acid in pure ethanol, store at -20°C. |
| **PAA** | 25 mM | Ethanol | Dissolve Palmitic acid alkyne in pure ethanol, store at -20°C. |
| **Myriocin** | 1 mM | Methanol | Dissolve Myriocin in methanol, store at -20°C. |
| **L-serine (1-13C)/(1-12C)** | 1M | Water | Dissolve 250 mg L-serine or ^13^C isotope-labeled serine in super clean water, dry, and resuspend in 2.361 mL or 2.339 mL super clean water. Store at -20°C. |

**Table S6. Whole-genome sequencing data availability**

| **Isolate name** | **SRA accession** | **BioSample accession** |
| --- | --- | --- |
| S6-C1 | SRR26987610 | SAMN38500364 |
| S6-C2 | SRR26987609 | SAMN38500365 |
| S6-C3 | SRR26987598 | SAMN38500366 |
| S6-C8 | SRR26987587 | SAMN38500367 |
| S6-C12 | SRR26987576 | SAMN38500368 |
| S6-D1 | SRR26987565 | SAMN38500369 |
| S6-D2 | SRR26987554 | SAMN38500370 |
| S6-D7 | SRR26987550 | SAMN38500371 |
| S6-D10 | SRR26987549 | SAMN38500372 |
| S6-E7 | SRR26987548 | SAMN38500373 |
| S6-E10 | SRR26987608 | SAMN38500374 |
| S6-E12 | SRR26987607 | SAMN38500375 |
| S6-F1 | SRR26987606 | SAMN38500376 |
| S6-F9 | SRR26987605 | SAMN38500377 |
| S6-F11 | SRR26987604 | SAMN38500378 |
| S6-G6 | SRR26987603 | SAMN38500379 |
| S6-G7 | SRR26987602 | SAMN38500380 |
| S6-G8 | SRR26987601 | SAMN38500381 |
| S6-H5 | SRR26987600 | SAMN38500382 |
| C6-A1 | SRR26987599 | SAMN38500383 |
| C6-A2 | SRR26987597 | SAMN38500384 |
| C6-B1 | SRR26987596 | SAMN38500385 |
| C6-B3 | SRR26987595 | SAMN38500386 |
| C6-B8 | SRR26987594 | SAMN38500387 |
| C6-D1 | SRR26987593 | SAMN38500388 |
| C6-D3 | SRR26987592 | SAMN38500389 |
| C6-F5 | SRR26987591 | SAMN38500390 |
| C6-H5 | SRR26987590 | SAMN38500391 |
| C6-H7 | SRR26987589 | SAMN38500392 |
| F2-B8 | SRR26987588 | SAMN38500393 |
| F2-A2 | SRR26987586 | SAMN38500394 |
| F2-C5 | SRR26987585 | SAMN38500395 |
| F2-H6 | SRR26987584 | SAMN38500396 |
| F2-C12 | SRR26987583 | SAMN38500397 |
| F2-A9 | SRR26987582 | SAMN38500398 |
| F2-C6 | SRR26987581 | SAMN38500399 |
| F2-E7 | SRR26987580 | SAMN38500400 |
| F2-H7 | SRR26987579 | SAMN38500401 |
| F2-D11 | SRR26987578 | SAMN38500402 |
| F2-A11 | SRR26987577 | SAMN38500403 |
| F2-C9 | SRR26987575 | SAMN38500404 |
| F2-E11 | SRR26987574 | SAMN38500405 |
| F2-H9 | SRR26987573 | SAMN38500406 |
| F2-F2 | SRR26987572 | SAMN38500407 |
| F2-A12 | SRR26987571 | SAMN38500408 |
| F2-C10 | SRR26987570 | SAMN38500409 |
| F2-F5 | SRR26987569 | SAMN38500410 |
| F2-H10 | SRR26987568 | SAMN38500411 |
| F2-F6 | SRR26987567 | SAMN38500412 |
| F2-B4 | SRR26987566 | SAMN38500413 |
| F2-C11 | SRR26987564 | SAMN38500414 |
| F2-H1 | SRR26987563 | SAMN38500415 |
| F2-H2 | SRR26987562 | SAMN38500416 |
| F2-B5 | SRR26987561 | SAMN38500417 |
| F2-D2 | SRR26987560 | SAMN38500418 |
| F2-H11 | SRR26987559 | SAMN38500419 |
| F2-B12 | SRR26987558 | SAMN38500420 |
| F2-D5 | SRR26987557 | SAMN38500421 |
| F2-H3 | SRR26987556 | SAMN38500422 |
| F2-H8 | SRR26987555 | SAMN38500423 |
| F2-C1 | SRR26987553 | SAMN38500424 |
| F2-D6 | SRR26987552 | SAMN38500425 |
| F2-H5 | SRR26987551 | SAMN38500426 |
